# P-1240. Precision in the Cradle: Evaluation of Cefepime Exposures in Neonates using Monte Carlo Simulations

**DOI:** 10.1093/ofid/ofaf695.1432

**Published:** 2026-01-11

**Authors:** Katie B Olney, Joel I Howard, David S Burgess

**Affiliations:** University of Kentucky HealthCare, Lexington, KY; UK Healthcare, Lexington, Kentucky; University of Kentucky, Lexington, KY

## Abstract

**Background:**

Although cefepime (CFP) dosing has been evaluated in neonatal pharmacokinetic studies, variations in pharmacodynamic targets, patient characteristics, and sampling times across such studies make interpretation of findings challenging.

**Figure:**
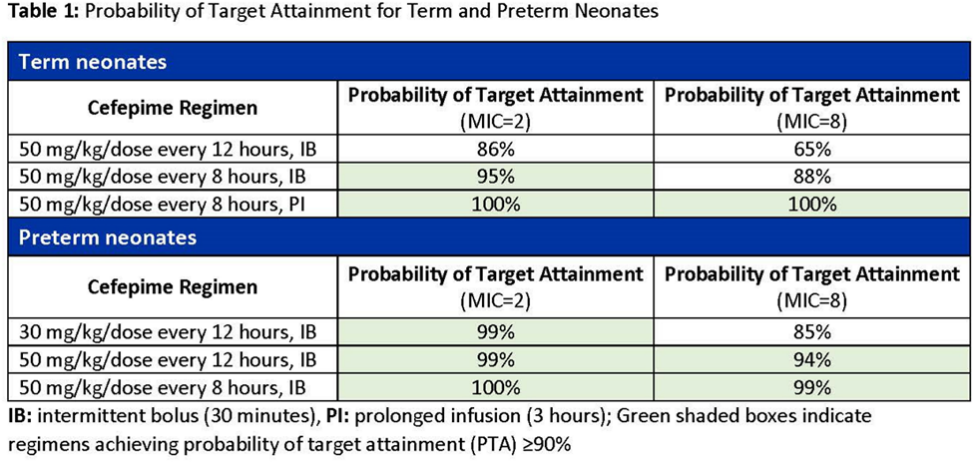

**Methods:**

We aimed to identify CFP dosing regimens that achieved at least 90% probability of target attainment (PTA) in premature and term neonates. Population pharmacokinetic parameters were collected from four published studies and stratified by gestational age. The pharmacodynamic efficacy target was 70%*f*T >MIC. Monte Carlo simulations were performed to evaluate the PTA associated with four CFP dosing regimens, stratified by gestational age and weight. MICs of 2 and 8 mg/L were selected to reflect CLSI susceptibility breakpoints for Enterobacterales (< 2 mg/L) and *P. aeruginosa* (< 8 mg/L), with 4-8 mg/L representing the susceptible-dose dependent (SDD) range for Enterobacterales. Achievement of PTA ≥90% at these MICs was considered optimal.

**Results:**

PTAs for specific regimens and MICs are displayed in Table 1. In term neonates, CFP 50 mg/kg q12h (intermittent bolus [IB]) achieved 86% PTA at an MIC of 2 mg/L, while 50 mg/kg q8h (IB) and 50 mg/kg q8h with prolonged infusion (PI) achieved 95% and 100% PTA, respectively. At 8 mg/L, the same regimens achieved PTA rates of 65%, 88%, and 100%, respectively, indicating that prolonged infusion is particularly important when targeting higher MICs. In preterm neonates, CFP 30 mg/kg q12h (IB) achieved 99% PTA at an MIC of 2 mg/L and 85% at 8 mg/L. CFP 50 mg/kg q12h (IB) achieved 99% PTA at an MIC of 2 mg/L and 94% at 8 mg/L. Dosing at 50 mg/kg q8h (IB) achieved 100% PTA at an MIC of 2 mg/L and 99% at 8 mg/L.

**Conclusion:**

Term neonates require higher CFP dosages (50 mg/kg/dose q8h) and prolonged infusions to achieve the pharmacodynamic target against a range of susceptible MICs. In preterm neonates, lower dosages (30 mg/kg/dose) achieved sufficient PTA only when the MIC was 2 mg/L; however, higher and more frequent dosing is necessary to achieve optimal PTA at an MIC of 8 mg/L. These findings are clinically relevant given the MIC susceptible breakpoint for Enterobacterales (<2 mg/L) and *P. aeruginosa* (<8 mg/L), with 4-8 mg/L representing the SDD category for Enterobacterales.

**Disclosures:**

All Authors: No reported disclosures

